# The therapeutic potential of losartan in lung metastasis of colorectal cancer

**DOI:** 10.17179/excli2020-2093

**Published:** 2020-06-29

**Authors:** Milad Hashemzehi, Niloufar Naghibzadeh, Fereshteh Asgharzadeh, Asma Mostafapour, Seyed Mahdi Hassanian, Gordon A. Ferns, William C. Cho, Amir Avan, Majid Khazaei

**Affiliations:** 1 Department of Medical Physiology, Faculty of Medicine, Mashhad University of Medical Sciences, Mashhad, Iran; 2 Student Research Committee, Faculty of Medicine, Mashhad University of Medical Sciences, Mashhad, Iran; 3 Metabolic Syndrome Research Center, Mashhad University of Medical Sciences, Mashhad, Iran; 4 Brighton & Sussex Medical School, Division of Medical Education, Falmer, Brighton, Sussex BN1 9PH, UK; 5 Department of Clinical Oncology, Queen Elizabeth Hospital, Kowloon, Hong Kong, China; 6 Department of Medical Genetics and Molecular Medicine, Faculty of Medicine, Mashhad University of Medical Sciences, Mashhad, Iran

**Keywords:** Renin-angiotensin system, losartan, colorectal cancer, lung metastasis

## Abstract

Colorectal cancer (CRC) is a common cancer with a high incidence rate. Components of the renin-angiotensin system (RAS) have been reported to be dysregulated in several malignancies including CRC. Here, we have explored the potential anti-metastatic effects of a RAS inhibitor, losartan, in an experimental model of lung metastasis in CRC. A murine model of lung metastasis of CRC was used, which involved the intravenous injection of CT26 cells via a tail vein. Four experimental groups comprised: an untreated group; a group that received 5-FU which was administered intraperitoneally; a losartan group and a combination group that received 5-FU plus losartan. We evaluated the anti-inflammatory effects of losartan by histopathological method, and the measurement of oxidative or antioxidant markers including malondialdehyde (MDA) and total thiol (T-SH) tissue levels, superoxide dismutase (SOD) and catalase activity. We found that losartan inhibited lung metastasis of CRC and there was a reduction of the IL-6 expression level in the tissue sample. It was also associated with reduced levels of the anti-angiogenic factor vascular endothelial growth factor (VEGF). Furthermore, we found that losartan induced oxidative stress as assessed by an elevation of MDA level, reduction of T-SH, SOD and catalase activities in lung tissue. Our findings demonstrated that losartan ameliorates angiogenesis, inflammation and the induction of oxidative stress via angiotensin II type I receptor (AT1R). This may shine some lights on targeting the RAS pathway as a potential therapeutic approach in the treatment of metastatic CRC patients.

## Introduction

As one of the most common malignancies, colorectal cancer (CRC) constitutes approximately 9 % of all cancers and is the fourth most common cause of cancer-related morbidity and mortality globally (Haggar and Boushey, 2009[11]; Siegel et al., 2017[26]). Colon cancer usually metastasizes to the liver and lung (Mun et al., 2019[20]). Although the progress of CRC screening and surveillance have contributed to the reduction in colon cancer mortality, tumor recurrence and metastasis remain a common cause of treatment failure. Therefore, it is important to explore new therapies for CRC.

There is growing evidence that components of the renin-angiotensin system (RAS) are expressed in different types of cancer, including colon cancer (Godugu et al., 2013[10]). Not only involved in body fluid homeostasis, this system also has been reported to regulate tumor progression (Wang et al. 2008[30]; Ager et al., 2008[2]). Angiotensin converting enzyme (ACE) is an important component of this system, and is involved in the production of angiotensin II (Smith and Missailidis, 2004[27]). Angiotensin II is a key factor in the renin-angiotensin system that also gears with cell proliferation, angiogenesis and migration of tumor cell through the activation of angiotensin type-1 receptor (AT-1) (Neo et al., 2007[22]; Uemura et al., 2003[29]). Some studies showed that the use of renin-angiotensin system inhibitors (RASIs) can reduce tumor development in several cancer types (Deshayes and Nahmias, 2005[7]). Previous studies have demonstrated that ACE and ARB reduce liver metastasis from colon cancer by reducing tumor growth, angiogenesis yet induce apoptosis of cancer cells (Neo et al., 2007[22]; Koh et al., 2014[14]). Interestingly, it has also been shown that these drugs can reduce the risk of cancer incidence and improve outcomes in cancer patients (Mc Menamin et al., 2012[18]). In this study, we have evaluated the effects of losartan, as an angiotensin receptor blocker, on lung metastasis of CRC in an experimental animal model.

## Material and Methods

### Reagents and chemicals

5-fluorouracil (5-FU), hematoxylin-eosin (H&E), losartan, and superoxide dismutase (SOD), total thiol (T-SH), catalase (CAT) and malondialdehyde (MDA) assays were purchased from Sigma-Aldrich Chemical Co. (St Louis, MO, USA). Streptomycin (50 μg/mL), penicillin (50 IU/mL), fetal bovine serum (FBS) and Dulbecco modified Eagle medium (DMEM) were purchased from Gibco (Gaithersburg, MD, USA). 5-FU and losartan were dissolved in normal saline and distilled water, respectively.

### In vitro model

The CT‐26 cell line was purchased from Pasteur Institute, Tehran, Iran. It was grown in DMEM supplemented with heat-inactivated FBS (10 %) and streptomycin/penicillin (1 %). The cells were incubated at 37 °C with 5 % CO_2_. 

### In vivo model

Twenty-four male inbred BALB/c mice (6-8 weeks old / 20-25 g) were purchased from the Pasteur Institute (Tehran, Iran). The animals were kept under standard conditions which were approved by the Institute Animal Ethics Committee of the Mashhad University of Medical Sciences (humidity of 54 ± 2 %, temperature 22 ± 2 °C and 12 h light/dark cycle). Mice were injected with 2x10⁶ CT26 cells by intravenous tail injection and were then treated from 24 hours after the cell injection for 10 days (Li et al., 2017[15]). The animals were randomly divided into four groups as follows (n = 6 in each group): 1) a control group, 2) a 5-FU only group (treated with 5 mg/kg every other day, intraperitoneally (ip)) (Marjaneh et al., 2018[17]), 3) a losartan group (treated with 90 mg/kg/day, ip), 4) a combination group (treated with 5mg/kg 5-FU every other day, ip and losartan 90 mg/kg/day, ip). Finally, the animals were sacrificed and the lungs were removed. The lung tissues were weighed, a part of the tissue was fixed for histological evaluation and another part was put into Bouin's solution for evaluation of appearance symptoms. The rest of the lung was stored at -70 °C for the examination of oxidant,antioxidant agents and inflammatory cytokines.

### Histological evaluation

The lung tissue was removed and washed with physiological saline and a portion of that was fixed in 10 % formalin. Tissue samples were embedded in paraffin, after dehydration, 5 μm thick sections were cut using a microtome. H&E stained sections were examined under light microscopy (×40 magnification).

### Evaluation of the metastatic microscopic nodules in the lung 

The isolated lung tissue was placed into Bouin's solution, the metastatic nodules were examined using a stereomicroscope. The number of metastatic microscopic nodules in different groups in the lung of mice was evaluated.

### Evaluation of oxidant and antioxidant agents

The fresh lung tissue was stored at -80 °C, and 50 mg of tissue was homogenized in PBS (pH 7.4), then, the homogenized solution was centrifuged for 10 mins and the total thiol group, CAT, SOD, and MDA were measured.

#### Evaluation of MDA

MDA concentrations were measured as a lipid peroxidation biomarker in lung tissue. 2 mL of thiobarbituric acid solution (TBA) was added to 1 mL of a color with absorption at λmax= 535 nm (10 % solution of homogenized lung tissue solution. The solution was incubated for 45 mins in a boiling water-bath, then it was centrifuged. MDA reacts with TBA and generates Janero, 1990[13]).

#### Measurement of total thiol groups

DTNB reagent was used to measure thiol groups. This reagent reacted with the SH groups and it would produce a yellow complex (nitro-mercapto benzoate anion), that had an absorption at λmax= 412 nm (Sadeghnia et al., 2013[24]). The total thiol content was measured in tissue based on Ellman method using the following formula: The rate of thiol groups = (A2-A1-B) × (1.07.05)/ 13.6

#### Measurement of SOD

The measurement is consisted of SOD generation through pyrogallol autoxidation and inhibition of MTT superoxide-dependent revival to formazan. The reaction was stopped by the addition of dimethyl sulfoxide (DMSO), which helps to dissolve formazan and produce a stable color. Overall, the appropriate amount of homogenized tissue, pyrogallol, and MTT was appended into the wells and kept for 5 mins at 37 ° C in the dark. After that, it was quenched using DMSO, and analysis was performed at λmax= 630 nm (reference) and 570 nm (Madesh and Balasubramanian, 1998[16]). 

#### Measurement of CAT enzyme activity

The activity of the CAT in tissue was measured by the Aebi method at the absorption spectrum of λmax= 240 nm. Based on this protocol, we used hydrogen peroxide (30 mM) and phosphate buffer (50 mM). The reaction began with the addition of hydrogen peroxide and the change in absorbance was measured by spectrophotometer for 1 min at λmax= 240 nm (Aebi, 1984[1]).

### ELISA

Measurements of VEGF/VEGFR-1, IL-6 in lung tissue were performed using Zellbio ELISA kits (ZellBio GmbH, Berlin, Germany) according to the manufacturer's instruction.

### In silico and heat map analysis of losartan response signature

We applied Autodock and LigPlot+ software or R software to evaluate the association of losartan with oxidant/antioxidant factors, IL-6, and VEGF/VEGFR1 markers.

### Statistical analysis

All data are shown as mean ± SEM and analyzed by one way ANOVA test followed by LSD as the *post hoc* test. The data were calculated using SPSS v.20 statistical software (IBM, Chicago, IL, USA). The statistical difference was significant at P < 0.05.

## Results

### Effect of losartan on survival, body weight and lung weight

**Figure 1:**
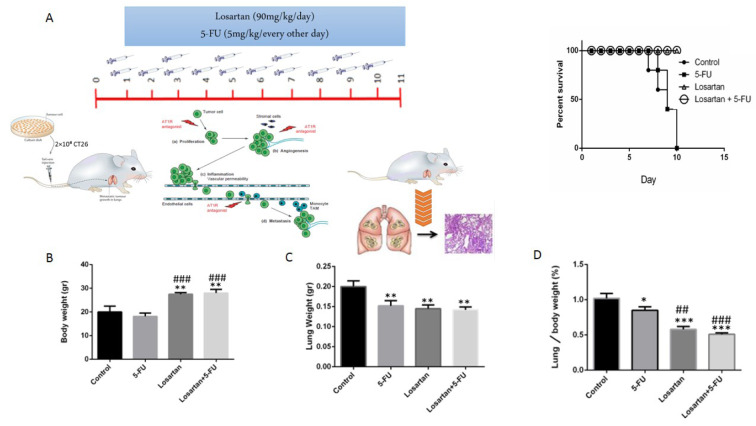
The treatment plan and survival rate of mice (A), Body weight (gr) (B), Lung weight (gr) (C), Lung to body weight ratio ( %) (D). Data are shown as Mean ± SEM (n = 6 per group). * P < 0.05, ** P < 0.01 and *** P < 0.001 compared with control group, ## P < 0.01 and ### P < 0.001 compared with 5-FU group.

Losartan alone and losartan plus 5-FU treatment groups increased the survival of the treated animals compared to the control and 5-FU groups (Figure 1A(Fig. 1)). We also assessed body weight at the end of the experiment. We observed a slightly decreased body weight in the 5-FU group, but it was not statistically significant. On the contrary, the body weights of the losartan and losartan+5-FU treated groups were significantly greater than those of the untreated group (P < 0.01) and the 5-FU group (P < 0.001) (Figure 1B(Fig. 1)). Furthermore, there was a significant reduction of lung weight in the 5-FU, losartan, and losartan+5-FU groups compared to the control group (P < 0.01), but in the losartan and combination group, compared with the 5-FU only group, there was no statistically significant difference (Figure 1C(Fig. 1)). In comparison to the controls, the percentage of the lung to body weight was lower in the 5-FU group (P < 0.05), as well as losartan and combination groups (P < 0.001 for both). This proportion in losartan and losartan+5-FU treated groups was statistically reduced compared with the 5-FU group (P < 0.01, P < 0.001 respectively) (Figure 1D(Fig. 1)).

### Histological and macroscopic assessment

**Figure 2:**
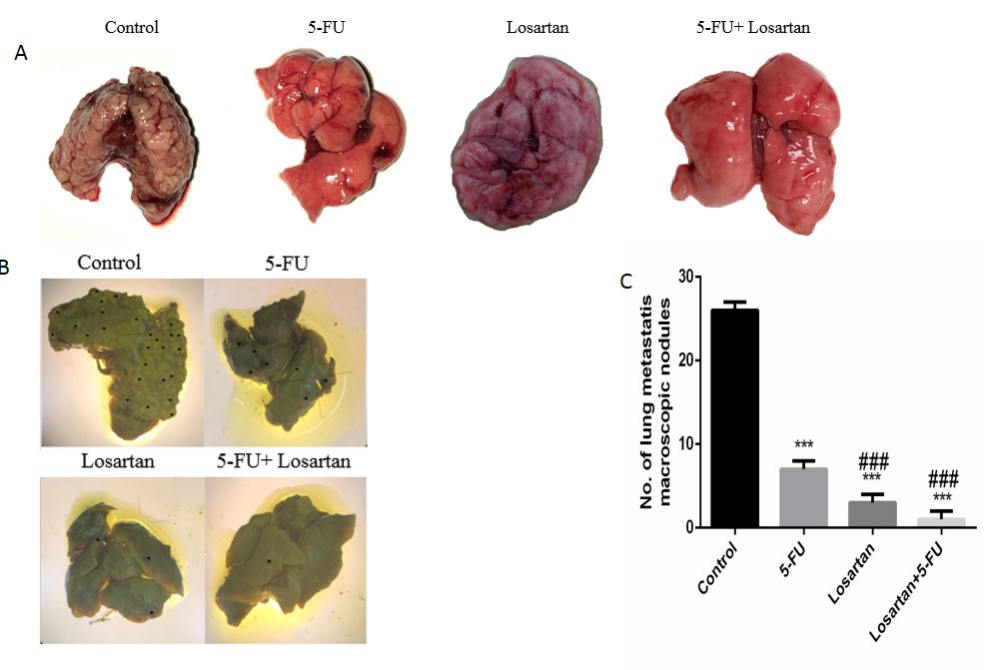
The number of macroscopic metastatic lung nodules (A-C). ***P < 0.001 compared to control group and ^###^ *P *< 0.001 compared to 5-FU.

**Figure 3:**
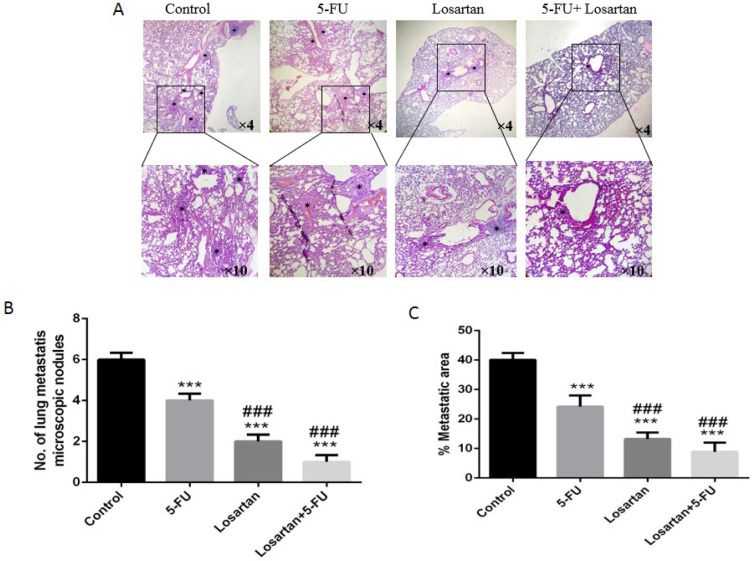
H&E staining demonstrated microscopic finding metastatic lung and foci in lung that are represented with black asterisks and the number of metastatic microscopic nodules in the lung of mice (A-B). The area of lung metastasis indicated was evaluated by an image analyzer (Image J, NIH, USA) (C). ***P < 0.001 compared to control group and^ ###^ *P *< 0.001 compared to 5-FU.

Intravenous injection of CT26 cells into mice led to the development of metastatic nodules in the lung tissue, but after treatment with 5-FU, losartan and the combination of losartan with 5-FU, metastatic nodules were significantly reduced (P < 0.001). Losartan and losartan+5-FU treatment reduced the number of metastatic nodules in lung tissue compared to the treatment with 5-FU (P < 0.001) (Figure 2A-C(Fig. 2)). Histological images show the number of microscopic nodules and percentage of metastatic area. These results showed that there was a significant decrease of microscopic nodules in 5-FU, losartan and co-administration of losartan with 5-FU treated groups, compared to control (P < 0.001). The number of microscopic nodules was significantly lower in losartan and losartan+5-FU groups compared to the 5-FU group (P < 0.001) (Figure 3A and B(Fig. 3)). Furthermore, histological evaluation also showed that 5-FU, losartan and losartan+5-FU reduced the percentage of metastatic area in tissue samples, compared to control group (P < 0.001). We also observed that percentage of metastatic area was reduced in losartan and losartan+5-FU treated groups compared to the 5-FU group (P < 0.001) (Figure 3C(Fig. 3)). 

### Oxidative and antioxidative parameters in lung tissue

**Figure 4:**
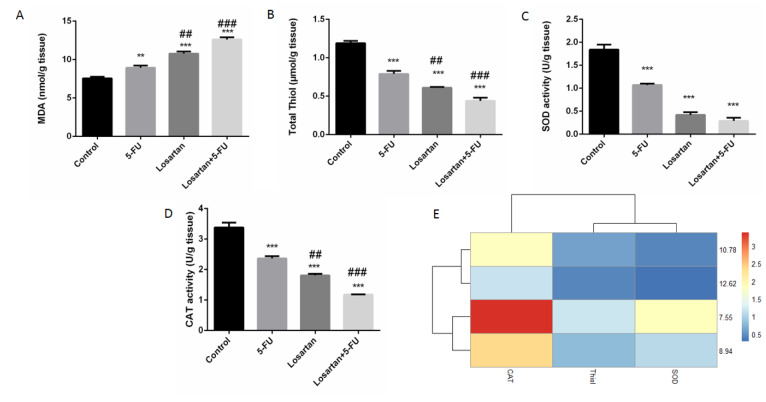
The MDA concentrations (A), SOD activity (B), CAT activity (C), total thiol contents (D) association between losartan and oxidant/anti-oxidant markers in tissue (E). Data are shown as mean ± SEM (n = 6 per group). ** P < 0.01 and *** P < 0.001 compared with control group, ^## ^P < 0.01 and ^###^ P < 0.001 compared with 5-FU group.

Statistically, we found that losartan and its combination with 5-FU increased MDA concentration, compared to the 5-FU treated group (P < 0.01 and P < 0.001, respectively) (Figure 4A(Fig. 4)). The level of MDA in the 5-FU group was higher than the control group (P < 0.01). The levels of SOD and CAT activities were significantly lower in all treated groups compared to the control group (P < 0.001 both of groups) (Figure 4C-E(Fig. 4)). CAT activity levels in the losartan and combination of losartan with 5-FU groups were significantly lower compared to the 5-FU group (P < 0.01 and P < 0.001, respectively). Furthermore, the total thiol content in all the treated groups was significantly lower than in the untreated group (P < 0.001 both groups). It has also been shown that total thiol content was significantly decreased in losartan and its combination with 5-FU than the 5-FU group (P < 0.01 and P < 0.001, respectively) (Figure 4B and E(Fig. 4)).

### IL-6, VEGF and VEGFR-1 levels

**Figure 5:**
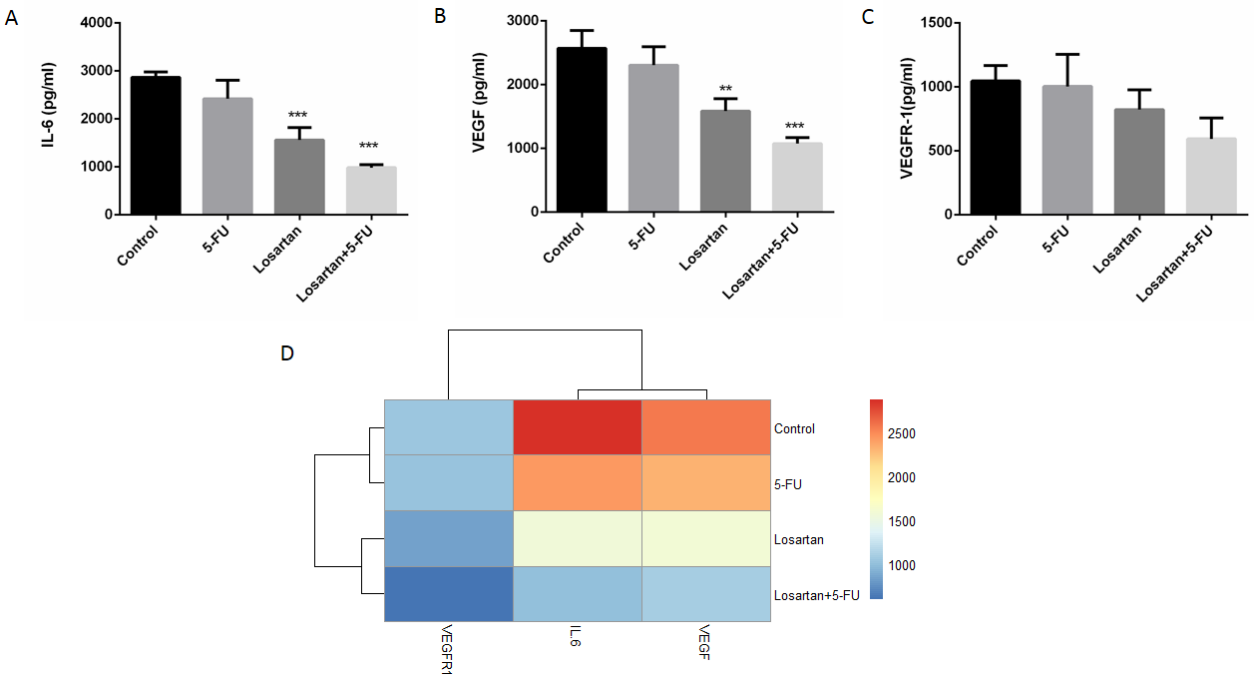
IL-6 level (A), VEGF level (B), VEGFR-1 level (C) and association between losartan and IL-6, VEGF and VEGFR-1 levels (D). Data are shown as mean ± SEM (n = 6 per group). ** P < 0.01 and *** P < 0.001 compared to control group.

The level of tissue IL-6 in the losartan and losartan+5-FU treated groups was significantly lower than the control group (P < 0.001), but IL-6 level in losartan and its combination with 5-FU treated groups was not significantly different compared to 5-FU treated group (Figure 5A and D(Fig. 5)). As shown in Figure 5B-D(Fig. 5), the levels of VEGF in the losartan group and the group treated with the combination of losartan and 5-FU were significantly lower compared to the control group (P < 0.01, P < 0.001 respectively). The VEGFR-1 level of all treated groups was not significantly different, compared to the control.

For more results see the Supplementary data.

## Discussion

To the best of our knowledge, this is the first study showing the anti-metastatic potential of losartan in CRC, yet in which lung metastasis is widely being reported (Chambers et al., 2002[5]; Neo et al., 2010[21]). It is well known that angiogenesis is crucial in cancer progression and is involved in the development of primary tumor, migration of tumor cells and distant metastasis (Harlozinska, 2005[12]). The ATII/AT1R pathway induces the formation of the new vessels. ATII activated MAPK and then increased VEGF expression (Fujita et al., 2002[9]). Previous studies have shown that RASIs reduced cancer cell metastasis by inhibiting angiogenesis through reduction of VEGF expression (Koh et al., 2014[14]; Miyajima et al., 2002[19]). Consistent with these results, we have demonstrated that losartan decreased angiogenesis through the reduction of VEGF level.

ROS production is related to a reduction of cell viability. It also induces cell apoptosis (Ahmadian et al., 2017[3]; Eftekhari et al., 2018[8]). Piskounova et al. demonstrated that oxidative stress suppressed metastasis of cancer cells (Piskounova et al., 2015[23]). In line with these results, Ahmadian et al. showed that Azilsartan, a novel blocker of AT1R, induced ROS formation and then activated apoptotic pathway (Ahmadian et al., 2018[4]). Interestingly, our results demonstrate that inhibitory effect of losartan on cancer cells metastasis correlates with oxidant/antioxidant status in tumor tissue.

There is some evidence that activation of AT1R in the tumor microenvironment is involved in progression of inflammation and metastasis (Deshayes and Nahmias, 2005[7]). ATII also enhances the expression of inflammatory cytokines such as IL-6 by activating AT1R (Suzuki et al., 2003[28]). Furthermore, Coulson et al. also demonstrated losartan could reduce tumor progression via down-regulation of inflammatory cytokine such as IL-6 (Coulson et al., 2017[6]). In addition, Sanchez et al. have shown that losartan decreases inflammation through reduction of IL-1/-6 and TNF-α levels in LPS-induced inflammation (Sánchez-Lemus et al., 2009[25]). For us, our results also demonstrated that losartan reduced IL-6 levels in tissue samples, compared to the control group.

In conclusion, our data has shown that losartan might reduce lung metastasis via down-regulation of angiogenic and inflammatory pathways in CRC; however, further extensive clinical studies may be warranted.

## Notes

Milad Hashemzehi, Niloufar Naghibzadeh and Fereshteh Asgharzadeh contributed equally as first author.

Amir Avan and Majid Khazaei (PhD, Metabolic Syndrome Research Center, Mashhad University of Medical Sciences, Mashhad, Iran; Tel: +98 513 8002298, E-mail: Khazaeim@mums.ac.ir) contributed equally as corresponding author.

## Funding

This study was supported by grants awarded by National Institute for Medical Research Development, Grant No. 976853 (Majid Khazaei).

## Disclosure

The authors have no conflicts of interest to declare.

## Supplementary Material

Supplementary data
