# Supplementary material for: The therapeutic potential of losartan in lung metastasis of colorectal cancer
Source: EXCLI J. 2020 Jun 29;19:927–35. doi: 10.17179/excli2020-2093 (PMC7355150; doi:10.17179/excli2020-2093)
Supplement: Supplementary data [file EXCLI-19-927-s-001.pdf]

EXCLI Journal 2020;19:927-935 – ISSN 1611-2156
Received: February 23, 2020, accepted: June 25, 2020, published: June 29, 2020

Supplementary data to:

THE THERAPEUTIC POTENTIAL OF LOSARTAN IN LUNG METASTASIS OF COLORECTAL CANCER

Milad Hashemzehi[1,2*], Niloufar Naghibzadeh[2*], Fereshteh Asgharzadeh [1,2*], Asma Mostafapour[3], Seyed Mahdi Hassanian[3], Gordon A. Ferns[5], William C. Cho[6], Amir Avan[2,3,4,#], Majid Khazaei[2,3,#]

[1] Department of Medical Physiology, Faculty of Medicine, Mashhad University of Medical Sciences, Mashhad, Iran
[2] Student Research Committee, Faculty of Medicine, Mashhad University of Medical Sciences, Mashhad, Iran
[3] Metabolic Syndrome Research Center, Mashhad University of Medical Sciences, Mashhad, Iran
[4] Department of Medical Genetics and Molecular Medicine, Faculty of Medicine, Mashhad University of Medical Sciences, Mashhad, Iran
[5] Brighton & Sussex Medical School, Division of Medical Education, Falmer, Brighton, Sussex BN1 9PH, UK
[6] Department of Clinical Oncology, Queen Elizabeth Hospital, Kowloon, Hong Kong, China

* These authos contributed equally as first author.

\# **Corresponding authors:** Majid Khazaei, MD, Ph.D., and Amir Avan, Ph.D. Metabolic Syndrome Research Center, Mashhad University of Medical Sciences, Mashhad, Iran; Tel: +98 513 8002298; E-mail: Khazaeim@mums.ac.ir; avana@mums.ac.ir;

http://dx.doi.org/10.17179/excli2020-2093

This is an Open Access article distributed under the terms of the Creative Commons Attribution License (http://creativecommons.org/licenses/by/4.0/).

EXCLI Journal 2020;19:927-935 – ISSN 1611-2156
Received: February 23, 2020, accepted: June 25, 2020, published: June 29, 2020

Supplementary Table 1: Body weight, Lung weight, and Lung to body weight ratio in treatment groups

Group	Body weight (g)	Lung weight (g)	Lung to body weight ratio
**Control, Mouse 1**	15.53	0.18	0.01159047005795235
**Control, Mouse 2**	16.22	0.19	0.011713933415536375
**Control, Mouse 3**	16.33	0.17	0.01041028781383956
**Control, Mouse 4**	26.81	0.21	0.007832898172323759
**Control, Mouse 5**	25.22	0.25	0.009912767644726409
**5-FU, Mouse 1**	17.33	0.13	0.007501442585112522
**5-FU, Mouse 2**	19.0	0.17	0.008947368421052633
**5-FU, Mouse 3**	14.35	0.13	0.009059233449477353
**Losartan, Mouse 1**	29.3	0.14	0.00506168933881683
**Losartan, Mouse 2**	26.12	0.12	0.006846556584776481
**Losartan, Mouse 3**	28.6	0.16	0.005361930294906167
**Losartan, Mouse 4**	28.39	0.16	0.004750593824228029
**Losartan+5-FU, Mouse 1**	31.61	0.16	0.004778156996587031
**Losartan+5-FU, Mouse 2**	24.83	0.17	0.004594180704441041
**Losartan+5-FU, Mouse 3**	26.11	0.14	0.005594405594405594
**Losartan+5-FU, Mouse 4**	29.47	0.14	0.005635787249031349

5-FU, Fluorouracil; Conc, concentration; g, gram of tissue; Four experimental groups comprised: a group untreated with either 5-FU or losartan; a 5-FU group that received 5 mg/kg/every other day, administered intraperitoneally (ip); a losartan group received 90 mg/kg/day; ip; a combination group that received 5-FU (5 mg/kg/every other day; ip) plus (losartan 90 mg/kg/day; ip). [Raw data to Figure 1, main document]

Supplementary Table 2: Number of lung metastatic in treatment groups

Group	Number of lung metastatic
**Control, Mouse 1**	23.0
**Control, Mouse 2**	22.0
**Control, Mouse 3**	28.0
**5-FU, Mouse 1**	6.0
**5-FU, Mouse 2**	5.0
**5-FU, Mouse 3**	4.0
**Losartan, Mouse 1**	4
**Losartan, Mouse 2**	2
**Losartan, Mouse 3**	1
**Losartan, Mouse 4**	3
**Losartan+5-FU, Mouse 1**	0
**Losartan+5-FU, Mouse 2**	1

5-FU, Fluorouracil; Conc, concentration; g, gram of tissue; Four experimental groups comprised: a group untreated with either 5-FU or losartan; a 5-FU group that received 5 mg/kg/every other day, administered intraperitoneally (ip); a losartan group received 90 mg/kg/day; ip; a combination group that received 5-FU (5 mg/kg/every other day; ip) plus (losartan 90 mg/kg/day; ip). [Raw data to Figure 2, main document]

EXCLI Journal 2020;19:927-935 – ISSN 1611-2156
Received: February 23, 2020, accepted: June 25, 2020, published: June 29, 2020

Supplementary Table 3: Percent of metastatic area and Number of metastatic nodule in treatment groups

Group	Percent of metastatic area	Number of metastatic nodule
**Control, Mouse 1**	1.159047005795235	23.0
**Control, Mouse 2**	1.1713933415536375	22.0
**Control, Mouse 3**	1.041028781383956	21.0
**Control, Mouse 4**	0.7832898172323759	
**Control, Mouse 5**	0.9912767644726409	
**5-FU, Mouse 1**	0.7501442585112522	9.0
**5-FU, Mouse 2**	0.8947368421052633	8.0
**5-FU, Mouse 3**	0.9059233449477353	7.0
**Losartan, Mouse 1**	0.506168933881683	
**Losartan, Mouse 2**	0.6846556584776481	
**Losartan, Mouse 3**	0.5361930294906168	
**Losartan, Mouse 4**	0.4750593824228029	
**Losartan+5-FU, Mouse 1**	0.47781569965870313	
**Losartan+5-FU, Mouse 2**	0.4594180704441041	
**Losartan+5-FU, Mouse 3**	0.5594405594405595	
**Losartan+5-FU, Mouse 4**	0.5635787249031349	

5-FU, Fluorouracil; Conc, concentration; g, gram of tissue; Four experimental groups comprised: a group untreated with either 5-FU or losartan; a 5-FU group that received 5 mg/kg/every other day, administered intraperitoneally (ip); a losartan group received 90 mg/kg/day; ip; a combination group that received 5-FU (5 mg/kg/every other day; ip) plus (losartan 90 mg/kg/day; ip). [Raw data to Figure 3, main document]

Supplementary Table 4: Oxidative stress markers

Group	MDA	Thiol	SOD	CAT
**Control, Mouse 1**	7.13	1.15	2.01	3.25
**Control, Mouse 2**	7.58	1.16	1.89	3.19
**Control, Mouse 3**	7.94	1.27	1.62	3.71
**5-FU, Mouse 1**	8.36	0.88	1.01	2.25
**5-FU, Mouse 2**	9.26	0.76	1.12	2.51
**5-FU, Mouse 3**	9.22	0.74	1.08	2.35
**Losartan, Mouse 1**	10.38	0.61	0.29921	1.82
**Losartan, Mouse 2**	10.67	0.63	0.45	1.72
**Losartan, Mouse 3**	11.29	0.59	0.52	1.9
**Losartan+5-FU, Mouse 1**	12.03	0.41	0.41	1.15
**Losartan+5-FU, Mouse 2**	12.83	0.39	0.315696	1.21
**Losartan+5-FU, Mouse 3**	13	0.52	0.145053	1.19

Malon dialdehyde: MDA; Superoxide dismutase activity: SOD; Catalase activity: CAT; Total thiol group: Thio; 5-FU, Fluorouracil; Conc, concentration; g, gram of tissue; Four experimental groups comprised: a group untreated with either 5-FU or losartan; a 5-FU group that received 5 mg/kg/every other day, administered intraperitoneally (ip); a losartan group received 90 mg/kg/day; ip; a combination group that received 5-FU (5 mg/kg/every other day; ip) plus (losartan 90 mg/kg/day; ip). [(Raw data to Figure 4, main document]

EXCLI Journal 2020;19:927-935 – ISSN 1611-2156
Received: February 23, 2020, accepted: June 25, 2020, published: June 29, 2020

Supplementary Table 5: IL-6 levels in treatment groups

Group	OD	Conc. pg/mL	g tissue	Conc. pg/g tissue
**Control, Mouse 1**	0.68	114.40	0.04	2790.24
**Control, Mouse 2**	0.553	93.57	0.04	2599.22
**Control, Mouse 3**	0.545	92.26	0.03	3075.33
**Control, Mouse 4**	0.534	90.46	0.03	3015.20
Average	**0.578**	**97.67**		**2870.00**
**5-FU, Mouse 1**	0.626	105.54	0.07	1507.77
**5-FU, Mouse 2**	0.571	96.52	0.04	2413.10
**5-FU, Mouse 3**	0.566	95.70	0.04	2392.60
**5-FU, Mouse 4**	0.6	101.28	0.03	3376.00
Average	**0.59075**	**99.76**		**2422.37**
**Losartan, Mouse 1**	0.657	110.63	0.05	2212.56
**Losartan, Mouse 2**	0.558	94.39	0.08	1179.90
**Losartan, Mouse 3**	0.659	110.96	0.10	1109.56
**Losartan, Mouse 4**	0.621	104.72	0.06	1745.40
	**0.62375**	**105.18**		**1561.86**
**Losartan+5-FU, Mouse 1**	0.682	114.73	0.10	1147.28
**Losartan+5-FU, Mouse 2**	0.549	92.92	0.10	929.16
**Losartan+5-FU, Mouse 3**	0.5	84.88	0.10	848.80
**Losartan+5-FU, Mouse 4**	0.6	101.28	0.10	1012.80
Average	**0.58275**	**98.45**		**984.51**

5-FU, Fluorouracil; Conc, concentration; g, gram of tissue; OD, optical density; IL-6 measurements in lung tissue were performed using Zellbio ELISA kits [Raw data to Figure 5A, main document]

EXCLI Journal 2020;19:927-935 – ISSN 1611-2156
Received: February 23, 2020, accepted: June 25, 2020, published: June 29, 2020

Supplementary Table 6: VEGF levels in treatment groups

Group	OD	Conc. pg/mL	g tissue	Conc. pg/g tissue
**Control, Mouse 1**	0.379	84.42	0.04	2059.02
**Control, Mouse 2**	0.381	84.98	0.04	2360.51
**Control, Mouse 3**	0.437	100.62	0.03	3354.07
**Control, Mouse 4**	0.346	75.20	0.03	2506.70
Average	**0.38575**	86.31		**2570.07**
**5-FU, Mouse 1**	0.464	108.16	0.07	1545.21
**5-FU, Mouse 2**	0.396	89.17	0.04	2229.22
**5-FU, Mouse 3**	0.438	100.90	0.04	2522.53
**5-FU, Mouse 4**	0.391	87.77	0.03	2925.73
Average	**0.42225**	96.50		**2305.67**
**Losartan, Mouse 1**	0.447	103.42	0.05	2068.31
**Losartan, Mouse 2**	0.469	109.56	0.08	1369.51
**Losartan, Mouse 3**	0.502	118.78	0.10	1187.80
**Losartan, Mouse 4**	0.447	103.42	0.06	1723.59
	**0.46625**	108.79		**1587.30**
**Losartan+5-FU, Mouse 1**	0.372	82.46	0.10	824.64
**Losartan+5-FU, Mouse 2**	0.525	125.20	0.10	1252.05
**Losartan+5-FU, Mouse 3**	0.502	118.78	0.10	1187.80
**Losartan+5-FU, Mouse 4**	0.449	103.97	0.10	1039.74
Average	**0.462**	107.61		**1076.06**

5-FU, Fluorouracil; Conc, concentration; g, gram of tissue; OD, optical density; Four experimental groups comprised: a group untreated with either 5-FU or losartan; a 5-FU group that received 5 mg/kg/every other day, administered intraperitoneally (ip); a losartan group received 90 mg/kg/day; ip; a combination group that received 5-FU (5 mg/kg/every other day; ip) plus (losartan 90 mg/kg/day; ip). VEGF/VEGFR-1 measurements in lung tissue were performed using Zellbio ELISA kits according to the manufacturer's protocol. [Raw data to Figure 5B, main document]

EXCLI Journal 2020;19:927-935 – ISSN 1611-2156
Received: February 23, 2020, accepted: June 25, 2020, published: June 29, 2020

Supplementary Table 7: VEGFR level in treatment groups

Group	OD	Conc. pg/mL	g tissue	Conc. pg/g tissue
**Control, Mouse 1**	0.469	34.47	0.04	840.7
**Control, Mouse 2**	0.448	30.49	0.04	847.1
**Control, Mouse 3**	0.496	39.57	0.03	1319.1
**Control, Mouse 4**	0.474	35.41	0.03	1180.4
Average	**0.47175**	34.99		1046.8
**5-FU, Mouse 1**	0.422	25.58	0.07	365.4
**5-FU, Mouse 2**	0.615	62.09	0.04	1552.1
**5-FU, Mouse 3**	0.48	36.55	0.04	913.7
**5-FU, Mouse 4**	0.475	35.60	0.03	1186.7
Average	**0.498**	39.95		1004.5
**Losartan, Mouse 1**	0.621	63.22	0.05	1264.4
**Losartan, Mouse 2**	0.6	59.25	0.08	740.6
**Losartan, Mouse 3**	0.578	55.09	0.10	550.9
**Losartan, Mouse 4**	0.52	44.11	0.06	735.2
	**0.57975**	55.42		822.8
**Losartan+5-FU, Mouse 1**	0.836	103.89	0.10	1038.9
**Losartan+5-FU, Mouse 2**	0.626	64.17	0.10	641.7
**Losartan+5-FU, Mouse 3**	0.469	34.47	0.10	344.7
**Losartan+5-FU, Mouse 4**	0.472	35.03	0.10	350.3
Average	**0.60075**	59.39		593.9

5-FU, Fluorouracil; Conc, concentration; g, gram of tissue; OD, optical density; Four experimental groups comprised: a group untreated with either 5-FU or losartan; a 5-FU group that received 5mg/kg/every other day, administered intraperitoneally (ip); a Losartan group received 90 mg/kg/day; ip; a combination group that received 5-FU (5mg/kg/every other day; ip) plus (Losartan 90 mg/kg/day; ip). VEGF / VEGFR-1 measurements in lung tissue were performed using Zellbio ELISA kits according to the manufacturer's protocol. [Raw data to Figure 5C, main document]
